# Breathing at a Distance: Telemedicine’s Achievements and Pitfalls in Respiratory Disease Management—A Narrative Review

**DOI:** 10.3390/diagnostics16213574

**Published:** 2026-10-29

**Authors:** Maria Chianese, Giorgio Monteleone, Francesco Salton, Paola Confalonieri, Michael Hughes, Yukai Wang, Umberto Semenzato, Pietro Geri, Marco Confalonieri, Eugenio Capparelli, Barbara Ruaro

**Affiliations:** 1 Pulmonology Unit, Department of Medical Surgical and Health Sciences, Hospital of Cattinara, University of Trieste, 34149 Trieste, Italy; 2 IRCCS AOU Ss Antonio e Biagio e Cesare Arrigo di Alessandria, 15121 Alessandria, Italy; 3 Center for Interstitial and Rare Lung Diseases, Department of Pneumology, Ruhrlandklinik, University Hospital Essen, University of Duisburg-Essen, 45239 Essen, Germany; 4 Centre for Musculoskeletal Research, Division of Musculoskeletal and Dermatological Science, School of Biological Sciences, Faculty of Biological Medicine and Health, The University of Manchester, Manchester Academic Health Science Centre, Stopford Building, Oxford Road, Manchester M13 9PL, UK; 5 Department of Rheumatology, Northern Care Alliance NHS Foundation Trust, Salford Care Organisation, Salford M6 8HD, UK; 6 NIHR Manchester Biomedical Research Centre, Manchester University NHS Foundation Trust, Manchester M8 5RB, UK; 7 Department of Rheumatology and Immunology, Shantou Central Hospital, Shantou 515041, China; 8 Respiratory Medicine, Department of Cardiac, Thoracic, Vascular Sciences and Public Health, University of Padova, 35121 Padova, Italy; 9 Rheumatology Unit, ASST Ovest Milanese, Legnano Hospital, 20025 Legnano, Italy

**Keywords:** telemedicine, telemonitoring, respiratory diseases, interstitial lung disease, obstructive sleep apnea, chronic obstructive pulmonary disease, home mechanical ventilation, cystic fibrosis, digital health

## Abstract

**Background/Objectives:** Telemedicine (TM) has become an increasingly important tool in the management of respiratory diseases, offering the potential to improve access to care, support long-term monitoring, and reduce the burden of in-person visits. This narrative review aims to critically summarize the current evidence on the achievements and limitations of TM across major areas of respiratory medicine. **Methods:** A non-systematic search of PubMed and Scopus was performed for English-language studies published up to April 2026, using combinations of keywords related to TM, telemonitoring, and respiratory diseases. Relevant articles were selected based on their clinical relevance and contribution to understanding the role of TM in diagnosis, monitoring, and management, and additional studies were identified through manual screening of reference lists. **Results:** The TM has shown consistent benefit for objective, device-derived outcomes—most notably CPAP adherence in obstructive sleep apnea—and for process outcomes such as reduced travel burden and improved cost-effectiveness primarily demonstrated in obstructive sleep apnea and COPD, with limited systematic evidence of cost-effectiveness in interstitial lung disease, home mechanical ventilation, cystic fibrosis, and smoking cessation. However, evidence for hard clinical endpoints such as hospitalization and mortality remains inconsistent, and successful implementation depends heavily on the surrounding care model rather than on technology alone. **Conclusions:** The TM represents a valuable complement to conventional respiratory care with demonstrated achievements in adherence, patient satisfaction, and healthcare efficiency, but its adoption remains limited by heterogeneous evidence, workforce and resource constraints, and the risk of widening digital disparities. Larger, adequately powered trials with harmonized outcomes and sustained investment in integrated care pathways are needed to fully realize its potential.

## 1. Introduction

Telemedicine (TM) represents an emerging technological approach in healthcare whose use has increased significantly over recent years [1,2]. It is defined as the delivery of healthcare services through modern information technologies to patients in remote or otherwise isolated settings [3,4]. Since its introduction, a growing body of evidence has explored its clinical applications, highlighting both potential benefits and limitations [5]. Its integration into routine healthcare has progressively required addressing concerns from healthcare professionals, institutions, and patients [5,6].

A pivotal acceleration in its global adoption occurred during the COVID-19 pandemic, when reduction of in-person contact became essential [7]. In this context, TM was widely implemented as a supportive tool in settings where face-to-face care was not feasible, including psychoeducation, rehabilitation, mental health support, and infectious disease management [1]. Although this period substantially expanded its use, important challenges remain for its sustainable implementation across healthcare systems [8].

Key barriers include socioeconomic disparities, limited digital literacy, regulatory heterogeneity, data privacy concerns, and challenges in standardization and continuity of care [9]. Additional limitations relate to cost-effectiveness, insufficient expertise, system design variability, and the absence of standardized protocols [10], alongside ongoing demands for technological updates, cybersecurity, and professional training [11,12].

Despite these limitations, TM has demonstrated clinical value in several domains. It is increasingly used for prognostic and monitoring purposes, including diabetic retinopathy grading, dermatologic assessment, and home monitoring of chronic respiratory diseases such as idiopathic pulmonary fibrosis (IPF) and chronic obstructive pulmonary disease (COPD) [3,4,6]. In COPD, telehealth integrated with telemonitoring may improve post-exacerbation outcomes, including quality of life and healthcare utilization [13,14].

In interstitial lung diseases (ILD), home monitoring strategies such as home spirometry provide valuable longitudinal data for both clinical management and research applications [15]. Similarly, TM-based telerehabilitation has shown non-inferior outcomes compared with conventional programs, with improvements in exercise capacity, quality of life, and adherence [16,17,18].

In asthma care, telemonitoring supports continuous disease management and multidisciplinary coordination, as highlighted by the COMETA consensus [19]. In obstructive sleep apnea (OSA), TM enables a shift toward decentralized, patient-centered care through remote diagnostics, teleconsultation, and telemonitoring of positive airway pressure therapy, with comparable short-term outcomes to standard care [20].

In patients receiving non-invasive ventilation, telemonitoring combined with education and support has shown potential benefits in improving hypercapnia control and therapy quality. Nevertheless, despite growing evidence of feasibility and clinical utility, important gaps remain regarding standardization, long-term effectiveness, equity of access, and real-world implementation [21]. Our narrative review critically evaluates the current evidence on TM in respiratory medicine, focusing on both its achievements and its limitations.

## 2. Materials and Methods

This narrative review was conducted to summarize and critically appraise the current evidence on the use of TM in respiratory medicine, with particular attention to its clinical applications, achievements, and limitations.

A non-systematic search of the literature was performed using PubMed and Scopus databases, focusing on studies published in English up to April 2026. The following keywords were used, alone and in combination: “telemedicine,” “telehealth,” “telemonitoring,” “respiratory diseases,” “COPD,” “asthma,” “interstitial lung disease,” “sleep apnea,” “non-invasive ventilation,” “cystic fibrosis,” and “smoking cessation.” Although “asthma” was included as a search term and yielded a substantial body of literature, a dedicated section on telemedicine in asthma was not included in this review because the identified studies were heterogeneous and their synthesis was considered beyond the focused scope of this narrative review; the topic is briefly acknowledged in the Introduction. Cystic fibrosis and smoking cessation were added as search terms after the initial search, following identification of clinically relevant evidence during manual reference screening.

Relevant articles were selected based on their clinical relevance to respiratory medicine and their contribution to understanding the role of TM in diagnosis, monitoring, and management of respiratory diseases. Additional studies were identified through manual screening of reference lists of selected articles and key reviews. Inclusion was not restricted by study design; articles were included if they provided clinically relevant evidence on TM applications, outcomes, feasibility, or implementation in the context of respiratory medicine.

Given the narrative nature of the review, no formal study selection criteria, risk of bias assessment, or statistical analysis was applied. The evidence was synthesized qualitatively, with the aim of identifying major themes, clinical applications, and current challenges in the implementation of TM in respiratory care. Throughout this review, “telemedicine” (TM) is used as an umbrella term encompassing telemonitoring (remote collection and transmission of physiological or device data), telerehabilitation (remotely delivered exercise or rehabilitation programmes), teleconsultation (synchronous or asynchronous remote clinical encounters), and mobile/eHealth interventions (smartphone applications and digital behaviour-change tools). Where the evidence pertains to a specific modality, the more precise term is used.

## 3. Telemedicine Application in Respiratory Diseases

### 3.1. Interstitial Lung Diseases

ILDs are a heterogeneous group of chronic and often progressive disorders characterized by inflammation and fibrosis of the pulmonary interstitium, frequently leading to significant morbidity and mortality [22]. Their management requires close longitudinal monitoring, including assessment of symptoms, pulmonary function, and imaging, ideally within a multidisciplinary setting [23,24]. Beyond follow-up, ILD care is strongly influenced by the complexity of early and accurate diagnosis, which is often delayed due to non-specific clinical presentation and the need for integration of radiological, functional, and sometimes histopathological data within multidisciplinary discussion. Over recent years, growing evidence has supported the role of digital health and TM approaches in the management of ILDs and related chronic cardiopulmonary conditions. One of the earliest RCTs in this field, conducted in patients with IPF, demonstrated that the integration of home monitoring with standard clinical care was associated with improvements in psychological well-being and quality of life over 24 weeks [25]. Building on this, subsequent studies have explored more technology-driven and patient-centered tools. Pilot data evaluating smartphone-based applications for remote monitoring in ILD populations have shown high adherence and sustained engagement, supporting their feasibility and acceptability in real-world settings [26]. In parallel, evidence from broader chronic disease populations indicates that structured telecare models—incorporating remote monitoring, symptom management, and coordinated multidisciplinary care—can improve patient-reported outcomes (PROMs), including quality of life [27]. Additional prospective data in IPF suggest that home-based spirometry, when supported by adequate training, may provide clinically meaningful insights into functional decline, disease progression, and patients’ prognosis [28,29]. In addition to innovations in monitoring, the therapeutic landscape of ILDs has evolved significantly with the introduction of antifibrotic agents such as nintedanib and pirfenidone, which have shifted management toward disease-modifying strategies aimed at slowing functional decline rather than only providing supportive care [30,31]. More recently, hybrid care models combining in-person and remote visits have emerged as a promising strategy to balance these needs. Randomized evidence in patients with myositis-associated ILDs receiving antifibrotic therapy suggests that such approaches are feasible and may support flexible, patient-centered follow-up while maintaining robust clinical oversight [32]. Furthermore, disease stratification into progressive fibrosing and non-progressive phenotypes has become increasingly relevant for guiding treatment decisions and follow-up intensity [33]. Nevertheless, despite the increasing adoption of TM, face-to-face visits remain the cornerstone of ILD management, particularly for comprehensive clinical evaluation, diagnostic accuracy, and multidisciplinary decision-making [34]. In contrast, clinicians generally express strong support for TM integration, recognizing its potential to enhance symptom monitoring, optimize care pathways, and improve efficiency [35]. This divergence in perspectives, widely reported in the literature, reflects differences in experience, expectations, and familiarity with digital health technologies. Moreover, TM-based strategies such as e-consultations, standardized referral pathways, and early identification of high-risk patients have also been associated with improved healthcare efficiency, including reduced waiting times and optimized access to specialist care [36]. Overall, TM is increasingly viewed as a complementary rather than substitutive component of ILD care; however, current evidence remains limited by heterogeneous study designs, relatively small sample sizes, and a lack of long-term outcome data. Additionally, patient selection remains a key consideration, as disease severity, digital literacy, and access to technology may significantly influence the effectiveness of remote care strategies.

### 3.2. Obstructive Sleep Apnoea

Continuous positive airway pressure (CPAP) remains the gold standard treatment for obstructive sleep apnoea (OSA), providing substantial improvements in daytime symptoms, quality of life, and cardiovascular outcomes in adherent patients [37]. Nevertheless, long-term adherence remains one of the major challenges in OSA management, as a considerable proportion of patients experience difficulties during treatment initiation and adaptation, resulting in suboptimal CPAP use [38]. This limitation has prompted growing interest in TM, particularly telemonitoring (TM), as a strategy to support patients during home therapy and optimize long-term disease management.

Telemonitoring enables the remote collection and transmission of PAP device data, including adherence, residual respiratory events, mask leaks, and pressure parameters, allowing healthcare professionals to identify and address technical or therapeutic issues promptly. This approach facilitates early interventions, such as mask replacement, humidification adjustment, or pressure optimization, potentially reducing treatment discontinuation while limiting the need for in-person consultations [39,40]. Furthermore, telemonitoring has become increasingly relevant in response to the growing prevalence of OSA and the imbalance between the rising demand for specialized sleep care and the limited availability of healthcare resources. In many healthcare systems, remote follow-up is already integrated with home care providers, who support patients technically while sharing device-generated data with sleep specialists through dedicated digital platforms. Multiphysician teleconsulting has similarly been proposed as a strategy to support CPAP prescription decisions in OSA [41].

Evidence supporting telemonitoring has steadily accumulated over the past decade. Numerous randomized controlled trials (RCTs) have evaluated different TM interventions, including remote monitoring, automated feedback systems, teleconsultations, and educational support. Although individual studies have reported variable results, several meta-analyses consistently demonstrate that telemonitoring improves CPAP adherence compared with usual care, with an average increase in nightly use of approximately 30 min [42,43,44,45]. The magnitude of benefit appears greater in systems based on wireless transmission of device data and in interventions combining remote monitoring with timely clinical feedback, suggesting that patient engagement and rapid problem-solving are key determinants of successful implementation [43]. Several studies have also shown that structured telemonitoring programs during the first three months of CPAP treatment—considered the critical period for establishing long-term adherence—are associated with improved compliance compared with conventional follow-up, although not all trials have reported statistically significant benefits [45,46,47,48,49].

Beyond adherence, telemonitoring has demonstrated additional advantages in terms of healthcare efficiency, patient satisfaction, and economic sustainability. Randomized studies have reported reductions in outpatient visits, travel requirements, and productivity losses, resulting in improved cost-effectiveness compared with conventional follow-up [50,51,52]. However, the available evidence remains heterogeneous, reflecting differences in TM interventions, healthcare organization, outcome definitions, and follow-up duration. Moreover, some earlier studies were limited by small sample sizes, heterogeneous patient populations, and insufficient statistical power to detect differences in patient-reported outcomes or long-term adherence [53,54,55,56].

Despite its demonstrated clinical value, the widespread implementation of telemonitoring faces important organizational challenges. Continuous surveillance of remotely transmitted data requires dedicated healthcare personnel, a resource that is often limited in sleep units already managing large patient populations [54]. As remote monitoring becomes more widely adopted, clinicians are increasingly confronted with large volumes of continuously generated data, raising concerns regarding information overload and the efficient prioritization of clinically relevant events [20]. Consequently, the future success of telemonitoring will depend not only on technological advances but also on the development of integrated care pathways, interoperable digital platforms, and intelligent decision-support systems capable of streamlining data interpretation and clinical workflow. In line with this perspective, the American Academy of Sleep Medicine has recently advocated a multidisciplinary, patient-centered model of care integrating sleep specialists, primary care providers, home care services, and patients to maximize the effectiveness and sustainability of TM in OSA management [55].

Italian experience is broadly consistent with these findings. A remote medical care centre model was shown to nearly double CPAP adherence among poorly compliant patients, with benefits persisting at 12 months [57], while a separate pilot programme delegating diagnostic titration and follow-up to an external telemonitoring centre working alongside local home-care providers proved feasible and improved compliance and daytime sleepiness in treatment-naïve patients [58]. Together with a recent national consensus document from the Italian Society of TM on the use of TM in OSA [59], these findings point to a growing, structured effort to integrate telemonitoring into routine sleep medicine practice, while underscoring the continued need for adequately resourced, standardized care models.

### 3.3. Home Mechanical Ventilation

Remote monitoring systems for long-term home mechanical ventilation (HMV) were first described roughly a decade ago, enabling continuous tracking of key ventilatory parameters—such as differential pressure, volume, and airflow—and the detection of clinical events including circuit disconnection and sleep apnoea across geographically dispersed patients [60,61]. Building on this early groundwork, the European Respiratory Society recently issued clinical practice guidelines on TM in HMV using an Evidence-to-Decision framework; given the generally low certainty of the underlying evidence, the initiation and follow-up of HMV via TM is only conditionally recommended for patients with neuromuscular or restrictive thoracic disease and for COPD, with no recommendation possible regarding which parameters should be monitored or for patients with obesity hypoventilation syndrome [62]. Consistent with this cautious but evolving picture, the multicentre, exploratory eVENT trial found that real-world telemonitoring and remote support for home non-invasive ventilation improved therapy effectiveness, reinforcing the potential of TM as an adjunct—rather than a replacement—for structured in-person follow-up [21].

### 3.4. Chronic Obstructive Pulmonary Disease

Optimal COPD management requires a holistic, integrated-care approach consistent with the Chronic Care Model, addressing the medical, social, psychological, and cognitive needs of patients across the disease trajectory—an approach that is resource-intensive to deliver conventionally. TM is a natural candidate to help meet this demand, with the most consistently favourable results reported for interventions targeting physical activity, non-invasive ventilator management, and telerehabilitation [63,64,65,66,67,68,69,70,71,72,73,74,75,76,77,78]. However, enthusiasm has historically outpaced supportive evidence: an interdisciplinary workshop held in Stresa in 2017 characterized TM in COPD as a technology “in need of a niche,” and a contemporaneous review concluded that considerably more evidence was required before TM could be regarded as genuine progress in COPD care [64,65,79,80,81,82,83,84,85,86]. An earlier systematic review of ten studies similarly found home telehealth to be broadly comparable to usual care for quality of life and satisfaction, with inconsistent effects on hospital bed-days and higher mortality in a telephone-support subgroup [66].

Large randomized evidence has echoed this cautious picture. The multicentre COMET trial, which added a home-based disease-management programme with weekly symptom questionnaires and case-manager oversight to usual care across 33 European centres, found no significant reduction in annualized unplanned hospitalization days, although secondary endpoints suggested modest benefit [67]. Similarly, an Italian RCT evaluating telemonitoring plus usual care found no significant improvement in health-related quality of life or hospitalization rates in a healthcare system with already well-established standards of care, suggesting that TM may be better targeted at specific patient subgroups rather than applied universally [87]. Reflecting on this evolving evidence base, subsequent commentary emphasized that successful implementation would require restructuring of clinical workflows, enhanced inter-professional communication, and sustained attention to patients’ digital literacy [68].

Evidence on hospital re-admission after acute exacerbation has been mixed: TM video consultation did not reduce cumulative length of re-admission stays compared with telephone follow-up or usual care in one trial, despite high patient satisfaction and sustained improvements in self-reported anxiety, depression, and symptom burden scores [69,70]. In contrast, telerehabilitation has emerged as a comparatively consistent area of benefit: two systematic reviews concluded that home-based telerehabilitation achieves improvements in exercise capacity, dyspnea, and quality of life that are at least non-inferior to conventional pulmonary rehabilitation, although optimal patient selection, delivery modality, and long-term maintenance strategies remain to be defined [71,72,73].

A separate and rapidly growing body of research has explored the use of digital monitoring and machine learning to detect physiological changes preceding acute exacerbations. Several studies support the ability of daily telemonitoring—of symptoms, respiratory mechanics, or respiratory rate—to detect deterioration before clinically apparent exacerbation onset [74,75,76,77,78], and a pooled analysis of two Danish RCTs found that telemonitoring significantly reduced moderate and severe exacerbations [79]. However, findings on hospital readmission remain inconsistent, with one RCT reporting no benefit of telemonitoring on readmission rates and another finding that adding a predictive algorithm to telemonitoring did not improve outcomes over telemonitoring alone [80,81]; a comprehensive review further concluded that methodological heterogeneity across studies continues to limit the generalizability of predictive digital tools in this field [82]. Table 1 summarizes the key studies in this area.

Italian pulmonology has similarly invested in TM for COPD. A national active home-surveillance system supported safe hospital-to-home transitions and early detection of clinical deterioration in a cohort of isolated patients [83], a knowledge-based telemonitoring application was developed to provide short- and long-term alerting based on physiological trends [84], and an AI-driven telemonitoring programme deployed in a geographically isolated region achieved a marked reduction in exacerbations and hospitalizations while overcoming barriers of distance and limited specialist availability [85]; the national scientific society has further estimated that telemonitoring of frequent exacerbators could meaningfully reduce the average annual direct cost of the disease [86,87]. Nonetheless, a national survey of experienced pulmonologists managing patients on long-term home non-invasive ventilation for chronic hypercapnic COPD found that telemonitoring uptake remained low (32%), constrained chiefly by insufficient human resources, absence of a structured regulatory framework, and reimbursement barriers, underscoring that technological readiness alone is not sufficient for successful implementation [88]. Table 2 summarizes these three Italian initiatives.

### 3.5. Cystic Fibrosis

Since the onset of the COVID-19 pandemic, TM has been increasingly explored in cystic fibrosis (CF) care. A narrative review highlighted its potential to enhance disease management by facilitating virtual consultations, supporting adherence to daily respiratory physiotherapy and exercise, enabling earlier detection of pulmonary exacerbations, and addressing patients’ psychological needs; however, the same review identified persistent limitations, including the inability to perform comprehensive physical examinations, reduced quality of in-person communication, and constrained microbiological surveillance—particularly relevant for the early detection of pathogens such as Pseudomonas aeruginosa, for which home sputum collection remains feasible but requires appropriate logistical infrastructure [89]. In the era of CFTR modulator therapy, characterized by improved survival and evolving long-term care needs, hybrid models combining remote and in-person services tailored to individual patients appear particularly promising. A complementary scoping review of telerehabilitation tools in paediatric and adult CF populations found considerable heterogeneity in study design and technology, limited use of CF-specific platforms, and restricted application of remote respiratory rehabilitation despite its clinical relevance, highlighting the need for more standardized, evidence-based technological development in this area [90].

### 3.6. Telemedicine in Smoking Cessation

Beyond disease-specific monitoring, digital health interventions—including text-messaging programmes, mobile applications, and broader eHealth platforms—have shown consistent benefit in supporting smoking cessation, particularly when incorporating personalized behavioural support and interactive engagement features [91,92,93,94,95]. However, the durability of these effects over the long term, and the clinical validity of non-personalized, standalone applications, remain important limitations, and substantial heterogeneity in intervention design and outcome assessment continues to constrain direct comparison across studies. Table 3 summarizes the main supporting studies.

### 3.7. Telemedicine in Italian Pulmonology: An Overview

This section is included because several disease-specific subsections above (OSA, COPD, ILD) draw substantially on Italian cohorts and surveys, and because Italy’s structured national investment in TM provides an instructive example of real-world implementation at system level. Beyond disease-specific applications, a broader picture of TM adoption among Italian pulmonologists has emerged from recent survey-based and policy-level work. Clinicians generally hold a favourable view of TM for patient education and monitoring alongside in-person care, yet actual implementation remains limited: national surveys report that TM is not uniformly available across regions, and that costs, staffing, and data-privacy requirements continue to constrain wider adoption [96,97]. A dedicated survey of the Italian Respiratory Society similarly found that although the large majority of respondents considered TM useful for ILD monitoring, only about one-third had implemented it in practice, citing limited service availability and insufficient familiarity with dedicated software as the main barriers [10]. At the regulatory level, recent national measures governing data protection within the national TM platform, together with position statements from the Italian Society of TM, reflect an evolving but still-maturing framework balancing interoperability, data security, and equitable access for patients with lower digital literacy [98,99]. Taken together, these findings suggest that—as in other national contexts—the successful scale-up of TM in Italian respiratory medicine will depend on coordinated investment in infrastructure, structured training, and standardized, adequately reimbursed care pathways.

### 3.8. Regulatory Considerations for Cross-Border Telemedicine in Europe

Beyond national experiences, a broader European regulatory framework is reshaping how cross-border TM can be implemented. Under the new European Health Data Space (EHDS) and Regulation (EU) 2025/327, artificial intelligence systems used in healthcare are classified as “high-risk” under the EU AI Act, requiring stringent standards of transparency, explainability, and human oversight [100]. The MyHealth@EU infrastructure is positioned as the cornerstone for health data exchange among Member States, with mandatory participation foreseen by 2029 for priority data categories such as patient summaries and electronic prescriptions, aiming to reduce fragmentation across healthcare systems while ensuring compliance with the General Data Protection Regulation (GDPR) [100]. Envisaged cross-border TM applications include support for international travellers, collaborative programmes between hospitals in different countries, remote reporting of radiological and histopathological images, and advanced interventions such as robotic telesurgery enabled by high-speed connectivity. Key enabling factors identified include EU funding mechanisms, technical interoperability through standardized data formats, agreed clinical protocols, and professional trust, whereas persistent barriers include legal and regulatory uncertainty, the absence of clear cross-border reimbursement schemes, language barriers, high infrastructure costs, and disparities in digital maturity across countries [100]. The long-term success of cross-border TM in respiratory (and other) disease areas will therefore depend as much on harmonized governance as on technological readiness.

## 4. Discussion

**Figure 1:**
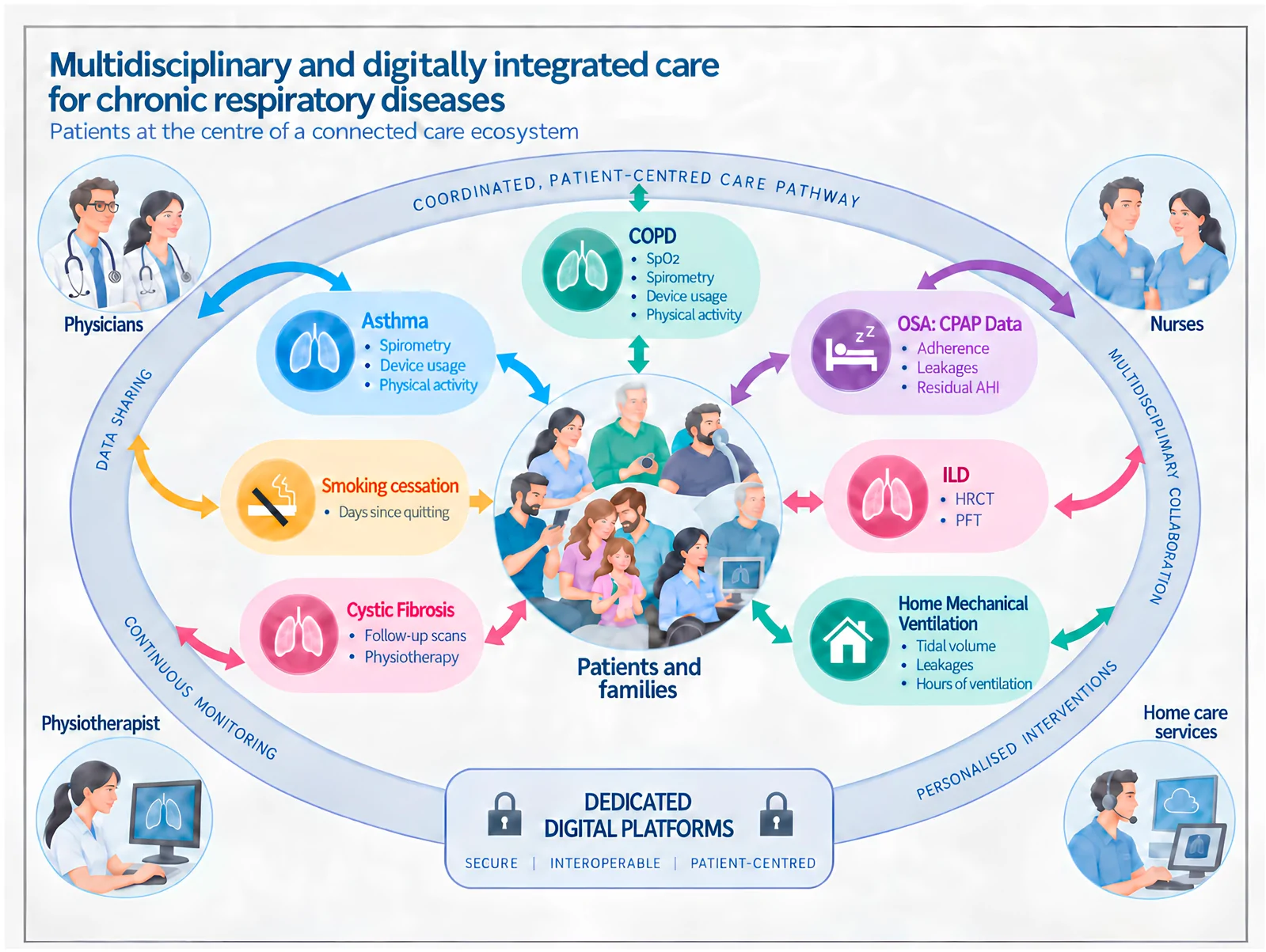
Telemedicine has shown measurable success in respiratory medicine by enhancing treatment adherence, increasing patient satisfaction, and optimizing healthcare efficiency across various diseases, while also ensuring continuity of care during times when face-to-face interactions are limited.

This narrative review highlights that TM has become an established, evidence-supported component of respiratory care across a wide range of conditions, from interstitial lung disease and obstructive sleep apnoea to home mechanical ventilation, COPD, cystic fibrosis, asthma and behavioural interventions such as smoking cessation. Across these diverse applications, several recurring themes emerge (Figure 1). It should be noted that the level of evidence varies substantially across disease areas: the strongest evidence derives from RCTs and meta-analyses (OSA, COPD telerehabilitation), whereas evidence in ILD, cystic fibrosis, asthma and smoking cessation relies largely on pilot studies and observational data. Feasibility and satisfaction outcomes are not equivalent to controlled evidence of clinical benefit.

First, the strength of evidence varies considerably by disease area and outcome. Telemonitoring shows the most consistent benefit for objective, device-derived outcomes—most notably CPAP adherence in OSA, where multiple meta-analyses converge on a clinically meaningful increase in nightly use—and for process-oriented outcomes such as reduced travel burden and improved cost-effectiveness. In contrast, evidence for hard clinical endpoints such as hospitalization and mortality remains inconsistent across ILD, COPD, and home mechanical ventilation, with several well-conducted RCTs (COMET, an Italian COPD trial, and a home-monitoring trial in IPF using the K-BILD questionnaire) failing to demonstrate superiority over usual care on their primary endpoints, despite consistent secondary benefits in patient-reported outcomes and psychological well-being.

Second, achievements and pitfalls are often two sides of the same technology. The same remote data streams that enable early detection of clinical deterioration and reduce unnecessary in-person visits also generate substantial data volumes that risk overwhelming already understaffed clinical teams, a concern raised consistently across the OSA, COPD, and home ventilation literature. Similarly, while TM can reduce geographic and logistical barriers to care, it may simultaneously widen disparities for patients with limited digital literacy or unreliable connectivity—a tension noted across virtually every disease area reviewed here, and particularly evident in real-world, non-trial settings such as those described in the Italian pulmonology experience.

Third, successful implementation appears to depend less on the sophistication of the technology itself than on the surrounding care model: structured multidisciplinary pathways, adequately staffed monitoring teams, clear escalation protocols, and sustained patient engagement consistently distinguish the studies reporting the greatest benefit. This is exemplified by hybrid models that position TM as a complement to, rather than a replacement for, in-person care, and by national initiatives that pair technology deployment with dedicated training and infrastructure investment.

This review has several limitations. As a narrative rather than a systematic review, study selection was not exhaustive, and the synthesis reflects qualitative judgment rather than formal quality or risk-of-bias assessment. Substantial heterogeneity across the underlying studies—in patient populations, TM interventions, outcome definitions, and follow-up duration—limits direct comparison and precludes quantitative synthesis. Much of the available evidence, particularly regarding real-world implementation, is drawn from single-country or single-centre experiences, which may limit generalizability to other healthcare systems.

## 5. Conclusions

TM has demonstrated tangible achievements across respiratory medicine, improving treatment adherence, patient satisfaction, and healthcare efficiency in several disease areas, while also enabling continuity of care during periods when in-person contact is constrained. However, its adoption remains hindered by persistent pitfalls—including heterogeneous evidence, unresolved questions about long-term clinical benefit, workforce and resource constraints, and the risk of widening digital disparities. Realizing the full potential of TM in respiratory disease will require larger, adequately powered trials with harmonized outcome measures, sustained investment in interoperable digital infrastructure, and care models that deliberately integrate technology within structured, adequately resourced clinical pathways rather than treating it as a stand-alone solution. In conclusion, although its use in the management of several respiratory diseases has yet to be fully investigated, the overall body of evidence supporting the utility of TM in clinical pulmonology is substantial and supports its implementation in the management of selected respiratory conditions in the near future. However, disease-specific protocols that are consistent, well-defined, and sufficiently detailed are still lacking. At the same time, the scientific and medical community should identify appropriate tools and strategies to facilitate the standardization of TM approaches across broader patient populations and disease categories.

## Figures and Tables

**Table 1:** Selected studies on digital monitoring and predictive tools for early detection of COPD exacerbations.

Study	Aim	Method	Main Finding
Miłkowska-Dymanowska et al. [74]	Feasibility of daily telemonitoring to predict acute exacerbations.	Pilot study; daily monitoring of symptoms and physiological parameters.	Monitored variables changed before exacerbation onset, supporting feasibility of early prediction.
Zimmermann et al. [75]	Whether day-to-day variability in respiratory mechanics enables early detection.	Home monitoring using the Forced Oscillation Technique (FOT).	Alterations in respiratory mechanics were detectable before clinical exacerbation onset.
Shah et al. [76]	Develop and validate a digital health system for exacerbation prediction.	Integrated platform combining symptom reporting, physiological monitoring, and predictive modelling.	Exacerbations identified and predicted with moderate accuracy, enabling earlier intervention.
Yañez et al. [77]	Whether home respiratory-rate monitoring enables early identification of exacerbations.	Continuous home monitoring of respiratory rate.	Respiratory rate increases preceded exacerbations by several days, supporting use as an early warning marker.
Colantonio et al. [78]	Develop decision-support models for personalized remote assessment.	Methodological study integrating remotely collected clinical data.	Demonstrated feasibility of intelligent systems for personalized monitoring and early deterioration detection.
Hyldgaard et al. [79]	Effect of telemonitoring on moderate/severe exacerbations.	Pooled analysis of two Danish RCTs comparing home telemonitoring with standard care.	Telemonitoring significantly reduced moderate and severe exacerbations.
Andersen et al. [80]	Effect of telemonitoring on readmissions after exacerbation.	RCT of post-discharge telemonitoring vs. usual care.	No significant reduction in hospital readmissions.
Kronborg et al. [81]	Whether an exacerbation-prediction algorithm improves outcomes over telemonitoring alone.	Comparative study; outcomes included hospitalizations and HRQoL.	No significant reduction in hospitalizations; only limited QoL improvement.
Konstantinidis et al. [82]	Role of digital tools in early diagnosis/prevention of exacerbations.	Comprehensive literature review of telemonitoring, wearables, mobile apps, and AI.	Considerable potential, but heterogeneous evidence insufficient for definitive recommendations.

**Table 2:** Selected Italian telemonitoring initiatives in COPD care: achievements, challenges, and mitigation strategies.

System	Achievements	Challenges	Mitigation Strategies
COD19 [83]	Ensured continuity of care for 226 isolated COPD patients; facilitated safe hospital-to-home transitions; enabled early identification of clinical deterioration through structured tele-surveillance.	High clinical complexity driven by advanced age and comorbidities; initial fragmentation of communication among GPs, hospitals, and regional health authorities.	Standardized discharge kit (pulse oximeter, thermometer, PPE); enrolment in active surveillance within 12 h via a centralized digital platform.
reCOVeryaID [84]	Short- and long-term early-alerting system based on physiological trends; optimized resource allocation by restricting hospitalization to unstable cases; high user satisfaction among clinicians and patients.	Risk of alarm fatigue from false positives on commercial devices; usability barriers for elderly or non-digital-native users.	Knowledge-based algorithm analyzing trend deviations rather than single-point thresholds; streamlined cross-platform web-app requiring no local installation.
Ogliastra experience [85]	Overcame geographical barriers to specialist care in rural/isolated settings; marked reduction in exacerbations and hospitalizations; enabled community-based diagnostics without patient travel.	Infrastructure-level digital divide and poor connectivity in mountainous regions; technological resistance among elderly populations; shortage of local specialists.	Predictive AI models for autonomous multi-parametric data analysis; digital hub-and-spoke framework with AI as a primary triage filter.

**Table 3:** Selected studies on digital and eHealth interventions for smoking cessation.

Study	Design	Main Finding	Limitations
Kinchen et al. [91]	Text-message (SMS) behavioral intervention.	Supported cessation via continuous behavioral support, reminders, and motivation.	Effectiveness may depend on message frequency, personalization, and adherence; long-term abstinence unclear.
Di Palo et al. [92]	Umbrella review of systematic reviews.	Digital modalities (apps, web-based, text messaging) generally showed positive effects and user engagement.	Considerable heterogeneity in intervention characteristics, outcomes, and follow-up across included reviews.
Shi et al. [93]	Systematic review and meta-analysis (multiple lifestyle behaviors).	eHealth interventions improved health-related behaviors; smoking outcomes improved when cessation was part of broader lifestyle programs.	Smoking cessation often a secondary outcome; substantial heterogeneity across studies.
Fang et al. [94]	Systematic review and meta-analysis.	eHealth interventions significantly increased abstinence; behavioral support and personalized feedback were most effective.	Significant heterogeneity in design and outcome assessment; risk of publication bias.
Cobos-Campos et al. [95]	Systematic review and meta-analysis.	Mobile applications showed potential benefit, particularly with evidence-based behavioral techniques.	Few high-quality RCTs; considerable variability in app content and outcome measures.

## Data Availability

No new data were created or analyzed in this study. Data sharing is not applicable to this article.

## Abbreviations

The following abbreviations are used in this manuscript:

TM	Telemedicine
CPAP	Continuous Positive Airway Pressure
OSA	Obstructive Sleep Apnoea
ILD	Interstitial Lung Disease
IPF	Idiopathic Pulmonary Fibrosis
COPD	Chronic Obstructive Pulmonary Disease
HMV	Home Mechanical Ventilation
NIV	Non-Invasive Ventilation
CF	Cystic Fibrosis
RCT	Randomized Controlled Trial
QoL	Quality of Life
HRQoL	Health-Related Quality of Life
K-BILD	King’s Brief Interstitial Lung Disease Questionnaire
AHI	Apnea–Hypopnea Index
PAP	Positive Airway Pressure
ERS	European Respiratory Society
AASM	American Academy of Sleep Medicine
SIP-IRS	Società Italiana di Pneumologia/Italian Respiratory Society
AGENAS	Agenzia Nazionale per i Servizi Sanitari Regionali
